# Christophe Terzian (1959-2016)

**DOI:** 10.1186/s13100-016-0068-6

**Published:** 2016-07-26

**Authors:** French Network on Mobile Genetic Elements

**Affiliations:** Université Clermont Auvergne, CNRS, INSERM, Laboratoire GReD, BP 10448, F-63001 Clermont-Ferrand, France

We learned with great sadness of the sudden death of our friend and colleague Christophe Terzian. He passed away at the age of 56 after a rapid and deadly disease.

Over the years Christophe played a major role in research on transposable elements successively in Lyon, Edinburgh, Clermont-Ferrand, Gif-sur-Yvette, Montpellier, Paris and back to Lyon where he had more recently been involved in the study of endogenous retroviruses from insects.

During his studies on transposable elements, he demonstrated with Alex Kim that Gypsy, a retrotransposon found in Drosophila melanogaster, indeed possessed infectious properties similar to mammalian retroviruses. What better gift for a passionately interested guy by Andalousia in Spain, to work on a transposable element controlled by a genomic locus called flamenco. He used to say "Flamenco makes gypsy dance". Flamenco has proved the soul of the party since it is now known to make many other transposable elements dance.

Christophe was respected for his fine mind and valued for his humor and his extreme kindness. He was also an accomplished teacher and a professor who was greatly appreciated by students and colleagues alike.

He will be greatly missed.

-------

We have opened a Google doc (testimony to Christophe Terzian [1]), for those who would like to post a message of testimony (in English, French or any other language). All the messages will be transmitted to Christophe’s family at the end of July 2016.
